# Kinematic analysis of work-related musculoskeletal loading of trunk among dentists in Germany

**DOI:** 10.1186/s12891-016-1288-0

**Published:** 2016-10-18

**Authors:** Daniela Ohlendorf, Christina Erbe, Imke Hauck, Jennifer Nowak, Ingo Hermanns, Dirk Ditchen, Rolf Ellegast, David A. Groneberg

**Affiliations:** 1 Institute of Occupational Medicine, Social Medicine and Environmental Medicine, Goethe-University, Theodor-Stern-Kai 7, Frankfurt am Main, 60590 Germany; 2 School of Dentistry, Department of Orthodontics, University Medical Centre of the Johannes Gutenberg University Mainz, Augustusplatz 2, Main, 55131 Germany; 3 Institute for Occupational Health and Safety (IFA) of the German Social Accident Insurance (DGUV), Alte Herrstraße 111, Sankt Augustin, 53757 Germany

**Keywords:** CUELA, Kinematic analysis, Dentist, Musculoskeletal disorder

## Abstract

**Background:**

In Germany, about 86.7 % of the dentists have stated to suffer from pain in the neck and shoulder region. These findings are predominantly based on surveys. Therefore the objective of this study is to conduct a kinematic analysis of occupational posture in dentistry.

**Methods:**

Twenty one dentists (11 f/10 m; age: 40.1 ± 10.4 years) have participated in this examination. The CUELA-System was used to collect kinematic data of the activities on an average dental workday. A detailed, computer-based task analysis took place parallel to the kinematic examination. Through the synchronization of data collected from both measurements, patterns of posture were arranged chronologically and in conjunction with the tasks performed: (I) “treatment” (II) “office” and (III) “other activities”. For the data analysis, characteristic data of joint angular distributions (percentiles P05, P25, P50, P75 and P95) of head, neck and torso at pre-defined tasks were examined and assessed corresponding to ergonomic standards.

**Results:**

Forty one percent of tasks executed on an average dental workday can be categorized as the treatment of patients. These tasked are most frequently performed in “straight back” positions (78.7 %), whereas 20.1 % were carried out in a “twisted or inclined” torso posture, 1.1 % “bowed” and only 0.1 % “bowed and twisted/inclined to the side” upper body position. In particular, it can be observed that in the area of the cervical and thoracic spine the 75th and 95th percentile show worse angular values during treatment than during non-dental tasks. For the period of treatment (at a standardized dental chair construction), a seated position with a strong inclination of the thoracic spine to the right while the lumbar spine is inclined towards the left is adopted.

**Conclusion:**

The kinematic analysis of dentists illustrates typical patterns of postures during tasks that are essential to the dental treatment of patients. The postures in the area of the cervical and thoracic spine have higher angular values during treatment compared to other dental tasks. Consistently, appropriate ergonomic design measures to optimize the dental chair and equipment as well as integrated training in ergonomics as part of the study of dentistry to prevent musculoskeletal are recommended.

**Electronic supplementary material:**

The online version of this article (doi:10.1186/s12891-016-1288-0) contains supplementary material, which is available to authorized users.

## Background

In the area of dentistry, ergonomics came into public spotlight. This is mainly due to the fact that in recent years an increasing number of studies have demonstrated that increased pain pathologies, especially in the neck, shoulder and/or back area, are directly related to the working conditions of dentists [1–7]. These findings are for the most part based on surveys [3, 8–14]. One of the key factors for the development of muscular imbalances and related muscular problems is the unsuitable posture of dentists during work [5, 15]. Alghadir et al. [16] report that there is a high prevalence of work-related musculoskeletal disorders among dental professionals (85 % of the respondents) after they started to work, whereby age-, gender- and work-related links to work-related disorders could be detected.

No less important, these physical ailments may also be the cause for which the occupational activities can only be carried out under pain [12, 14, 17] or for which the dentists can no longer work in their profession at all [18, 19]. The musculoskeletal disorders of dentists are probably due to long working hours in static positions, mostly in incorrect working postures, without longer breaks, as well as to recurrent and repetitive movements [6, 12, 20].

The essential point of these studies is the fact that musculoskeletal pain impact daily working life. In order to reduce this type of pain, there is an increased demand for in-depth ergonomic studies to analyze the dentists’ daily work routine as such but also the working environment. Blanc et al. [21] have conducted an ergonomic study on different dental units and demonstrated various muscular activities with corresponding joint angles depending on the working postures at the workstation. The authors concluded that a dentist’s musculoskeletal strain is quantifiable, comparable, and especially very variable so that musculoskeletal disorders can be decreased by improving the ergonomic positioning of the patient and of the practitioner. In addition to ergonomic recommendations, Dajpratham et al. [7] as well as Gupta et al. [10] pointed to the implementation of a complementary medicine strategy or an incorporated alternative medicine strategy which promotes musculoskeletal health. This would, on the one hand, have a positive effect on the dentist’s career and, on the other hand, prevent musculoskeletal disorders [7, 10].

Most studies come to the conclusion that systematic ergonomic analyses of dentists’ working positions are missing and need to be conducted in the near future. Existing ergonomic analyses, such as the one by Blanc et al. [21], are predominantly sequential (orientation of the practitioner towards the patient during the treatment) and have a short-term focus. In other cases, Pirvu et al. [22] or Jodalli et al. [23] have presented theoretical explanations and analyses of the emergence of work-related musculoskeletal disorders in dentists. Despite this unified general belief, comprehensive kinematic analyses that focus on the dentist’s entire daily work routine and the respective body postures have not yet been conducted in the field of dentistry. The previous literature also shows that work stress as well as work load of dentists is high. Therefore, further studies should be carried out in future.

It is, therefore, the objective of this study to analyze the movements and body postures of dentists during their day-to-day work. To enhance the data analysis, the movement analysis will be combined with an objective activity analysis [24] and classified into the following three categories: (I) “treatment” (II) “office” and (III) “other activities”. This categorization ensures a detailed kinematic analysis of the movements that is related to treatment as well as to unspecified dental movements. The following hypotheses are to be tested in this study:

- Hypothesis 1: The category “treatment” represents the largest percentage of the overall working time of dentists.
- Hypothesis 2: Unfavourable body postures are adopted particularly during treatment.
- Hypothesis 3: Patterns of dentist specific posture become apparent in the category “treatment”.

## Methods

### Subjects

Twenty one dentists (11 f/10 m) with an average age of 40.1 ± 10.3 years and a work experience of 10.6 ± 9.9 years participated in this study. With regard to handedness, only one left-handed woman was among the subjects. All participants have successfully completed the study of dentistry at a German University and worked either as training assistants or dentist in an established practice. According to their own statements, none of the participants showed signs of functional impairment or ailments related to the musculoskeletal system. In addition, injuries of the musculoskeletal system occurred more than 2 years prior to the study.

All participants were registered by the Association of Dentists of Hessen (Germany) and listed in a publicly accessible register. This register concomitantly served as the means by which the dentists in the Frankfurt/Main area were contacted and, ultimately, by which participants of this study were selected. Subjects were asked to participate by an official letter addressed to the practice owner informing about the planned investigation. The letter contained the most basic information. Following their agreement to participate to the study, the dentists were personally informed about the goals and the approach of the study. Based on a previous sample size calculation in terms of flexion during dental activities 21 participants were determined.

This study was approved by the Ethics Committee (135/14) of Goethe University in Frankfurt am Main. All the participants signed in advance an informed consent required to take part in the study.

### Measuring system: CUELA

**Fig. 1:**
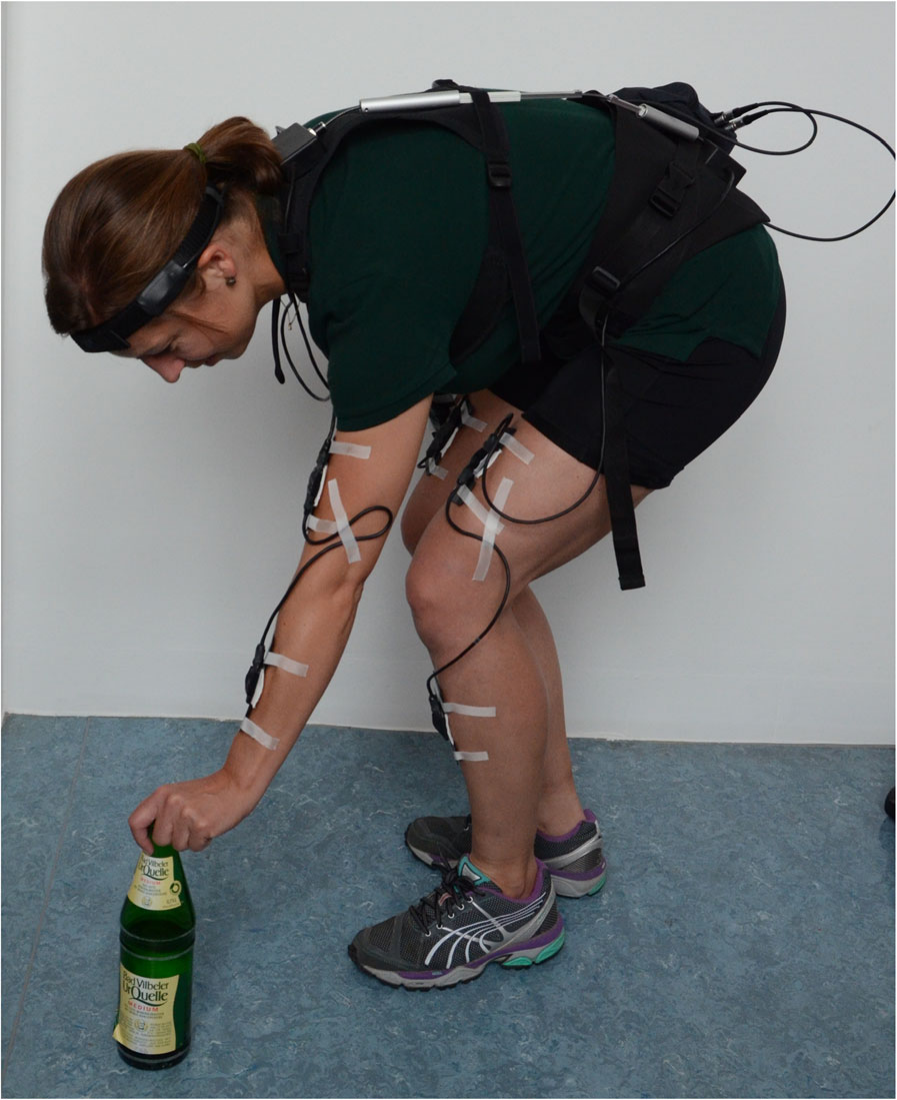
Illustration of the CUELA-System

The CUELA-System (computer-assisted acquisition and long-term analysis of musculoskeletal loads), developed at the Institute for Occupational Safety and Health of the German Social Accident Insurance (IFA; Sankt Augustin, Germany), was used to record body postures [25, 26]. This personal system uses sensors (accelerometers [ADXL 103/203] and gyroscopes [muRata ENC-03R] for head, arms, legs, back and potentiometers [Contelect] for back torsion) to measure the position or the angle and, in this way, enables a kinematic reconstruction of the movements of the subjects. The sensors were attached underneath clothing on arms, legs and head, as well as in the area of the thoracic and lumbar spine (Fig. 1) [27].

The possible degrees of freedom which accurately represent the dynamic movements are detected by the CUELA-System with a sampling frequency of 50 Hz and an angular resolution of approximately ±1°. The body angles, which have been measured or, respectively, calculated in the course of this study are listed in Table 1 [28–30].

**Table 1:** Depiction of the recorded body/joint angles based on the OWAS method, applied evaluation parameters and assessment criteria according to ergonomic standards

Body areas	Joint/body area	Assessed movementsaccording to medical definitions	Parameters to evaluate	Angle range values according to ergonomic standards
Head/neck	Head	sagittal inclination	Head tilted to the front (HT_f) [32, 42]	Neutral: 0 to 25° Moderate: 25 to 85° Awkward: < 0° & > 85°
		lateral inclination	Head tilted to the right (HT_r) 42]	Neutral: -10 to 10° Awkward: < -10° & >10°
	Cervical spine (CS)	flexion/extension	Neck curvature to the front (NC_f) *[Difference between head and TS]* [32, 42]	Neutral: 0 to 25° Awkward: < 0° & > 25°
		lateral flexion	Neck curvature to the right (NC_r) *[Difference between head and TS]* [32, 42]	Neutral: -10 to 10° Awkward: < -10° & >10°
Back	Thoracic spine (TS)	flexion/extension	TS inclination to the front (TSI_f) [32, 42]	Neutral: 0 to 20° Moderate: 20 to 60° Awkward: < 0° & > 60°
		lateral flexion	TS inclination to the right (TSI_ r) [32, 42]	Neutral: -10 to 10° Moderate: -10 to -20° Moderate: 10 to 20° Awkward: < -20° & > 20
	Lumbar spine (LS)	flexion/extension	LS inclination to the front (LSI_f)	No ergonomic layout available
		lateral flexion	LS inclination to the right (LSI_r)	
	Torso	flexion/extension	Back curvature to the front (BC_f) *[Difference between TS and LS]* [32, 42]	Neutral: 0 to 20° Moderate: 20 to 40° Awkward: < 0° & > 40°
			Inclination of the torso to the front (TI_f) *[Median flexion of TS and LS]* [32, 42]	Neutral: 0 to 20° Moderate: 20 to 60° Awkward: < 0°& > 60°
		lateral flexion	Back curvature to the right (BC_r) *[Difference between between TS and LS]* [32, 42]	Neutral: -10 to 10° Moderate: -10 to -20° Moderate: 10 to 20° Awkward: < -20° & > 20°
			Inclination of the torso to the right (TI_r) *[median lateral flexion of TS and LS]* [32, 42]	
		torsion	Back torsion to the right (BT_r) *[Difference between TS and LS]* [42]	

### Measuring system: activity objective analysis with mini-PC

A software that has been specifically developed for the analysis of activities enables real time recording of dentist performed procedures on a hand-held computer (UMPC Samsung Q1, Samsung Electronics GmbH, Schwalbach, Germany). For an accurate description of the performed dental activities, the software includes a spectrum of possible activity categories. On the one hand, this will allow for identification of the activity as such, whereas, on the other hand, the software can also determine the duration of these activities within the daily work routine. For a more detailed description of the system, please refer to the methods paper by Mache et al. [24, 31].

### Experimental procedure

For each participant, an average work day of a dentist is randomly selected. Within the scope of the kinematic analysis, sensors of the CUELA-System were attached to the participants’ arms, legs and head as well as to the spine. Parallel to the recording by the CUELA-System, observers assisted the participants and documented every movement of the dentist by means of task analysis on the hand-held computer. Prior to the experiment, the work behavior of dentists was documented through precise observations and analyses. The respective results were discussed and analyzed in collaboration with the dentists. These activities were subsequently implemented into the activity analysis software. The range of dental activities was divided into three categories: (I) “treatment” (II) “office” and (III) “other activities.” These categories represent 18 activities. Each activity corresponds to one of the many tasks involved in the day-to-day work of a dentist. For matters of coherence, these categories were simplified in order to group similar movement patterns and to enable a comparison thereof (Table 2).

**Table 2:** Depiction of all categories with the respective work stages, their explanation and the respective duration

Category	Activity	Details	Duration [min]
Treatment (I)	Impression	Taking an impression of the patient’s teeth	40.8
	Handicraft activities	Umbrella term for the following activities, respectively all work stages that are not included in the aforementioned activities.	1790.2
		Dental implant procedure: placing an implant	
		Tooth extraction: extracting a tooth	
		Pain diagnostic	
		Dental injection : using a syringe	
	Palpation	Palpation of TMJ and muscles of mastication	7.1
	Break	Short breaks during the treatment	53.0
	X-ray	Radiographic examination	26.4
	Screening	First screening / check-up of patients	255.1
	Dental handpiece	Using contra-angle/turbine/ultrasonic handpiece during the treatment	688.9
Office (II)	Treatment plan	Analysis and conception of treatment plans based on dental casts and X-rays (Arbeiten in der Akte/am Model am Schreibtisch)	4.3
	Phone calls	Having conversations on the phone	159.7
	Files/computer work	Medical record completion, whether in paper or electronic format	450.8
	Records	Reading patient records (results/dental casts/X-ray) (nur Akteneinsicht)	970.5
Other activities (III)	Meeting	Medical consultation among peers	99.3
	Conversation	Conversations with patients and staff as solitary activity	1693.9
	Hygiene	Hygienic measures (washing/disinfecting hands/ wearing gloves/face masks)	93.1
	Take/deposit instrument	Taking up instruments from a drawer/putting instruments down during and after the treatment	243.6
	Laboratory	Any kind of laboratory work	179.1
	Walk	Covering distances	230.4

### Evaluation

Synchronizing the activity analysis and the CUELA measurement in the CUELA software enables a temporal allocation of the motion patterns found in the individual activities of dentists. Due to the vast amounts of performed tasks, activities were preselected based on their relevance and on the percentage of the duration of the treatment of patients. To compare the measured joints angles of the different activities the percentiles 5, 25, 50 (Median), 75 and 95 (abbreviated as P05, P25, P50, P75 and P95) are used as indicators. For example, regarding the P05-value of an activity, 5 % of all measured angle values are below and 95 % are above.

The evaluation parameters specify the exact angle values for a particular body region. If a rotation, curvature or inclination is described in one direction (positive sign), the negative sign of the value refers to an opposite direction of the movement. This is particularly the case with lateral movements.

For every angular value of each body region (evaluation parameters), the percentile intervals were assigned to a color-coded angle range in accordance with ergonomic standards (traffic light: system red/yellow/green). Based on the respective colors, postures were assessed as unfavorable (awkward), moderate or neutral [32–34] (Table 1).

Moreover, the variance of the motion of a specific activity has to be taken into account. This was carried out by means of the modified interquantile range (mIR = [(P50-P05) + (P95-P50)]/2). To date, there is no economic layout available for the assessment of the mIR. The higher the value, the greater the variance of movement.

In addition, the Ovako Working posture Analysis System (OWAS) assesses body postures and movements with regard to their temporal share of the activity and estimates the resulting musculoskeletal loading [35, 36].

## Results

**Fig. 2:**
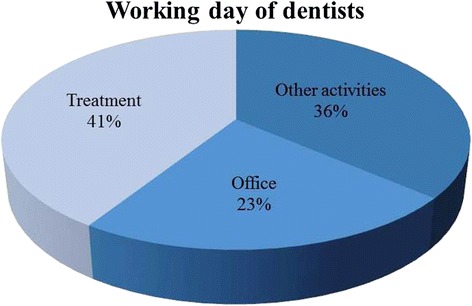
Depiction of the three categories with the respective percentage of their duration

The measurements generated 116.4 h (6986.4 min) of usable data material, exept the non-related activities (such as breaks or toileting). The amount of data is divided into the following three categories: 41 % (2861.5 min) belongs to category I “treatment”, 23 % (1585.2 min) to category II “office” and 36 % (2539.4 min) to category III “other activities” (Fig. 2a; Additional file 1: Table S1).

The category “treatment” (I) comprises seven activities. The two most important tasks taking up the largest amount of time are “handicraft activities” and “contra-angle/turbine/ultrasonic handpiece”. Taken together, these two activities account for 87 % (2479.2 min) of the overall treatment time.

In “office” (II), 90 % (1421.2 min) of the working time is taken up by entering data into files, respectively computer work and consulting files and findings as well as working on treatment plans. In the category “other activities” (III), the task “conversation” (67 %) (1693.9 min) occupies the largest percentage of time.

The following descriptive specification of the respective sub-activity refers to all percentiles as well as the mIR of Additional file 1: Table S1, Additional file 2: Table S2 and Additional file 3: Table S3.

### Treatment (I)

Additional file 1: Table S1 comprises the percentile values as well as the modified interquantile range of category I. In the *head and neck area*, the P50-data varies with the head tilted to the front (HT_f) between 2° - 45° (mIR: 14° - 26°) with all activities being in the neutral or in the moderate range. The P75 and P95 angle values lie between 9° - 57°. For the head tilted to the right (HT_r), the P50 values are between -1° and 10° (mIR: 10.5° - 23°) and P05 and P95 show angle values between -17°–-9°, respectively 8° - 32°. While the P25-P75 values are predominantly in the neutral range, the P05 and P95 values are mostly in the unfavourable range. These lateral movements indicate a stronger inclination to the right, whereby the value of P95 (32°) is almost twice as high as the P5 (-17°) value to the left.

With regard to the neck curvature to the front (NC_f), it becomes apparent that most sub-tasks at P50 and P75 can be found in the neutral range (-6°–26°), whereas the P95 angle values lie predominantly in the unfavorable range (12° - 34°). The mIR lies between 11.5° to 21.5°.

Similar assessments can be made regarding the P50 values of the neck curvature to the right (NC_r) as all of them lie in the neutral range (-5°–5°; mIR: 9°–16°). The angles of the P95 values (5°–19°) as well as the angles of P05 (-8° to -20°) are mainly in the unfavourable range. Neck curvatures during treatment activities are thus predominantly symmetrical (negative values represent neck curvatures to the left while positive values denote those to the right). According to the percentile values, the dimension of the movements (value of data) is almost identical.

In the *torso* area, the values of the TS inclination to the front (TSI_f) at P75 and P95 are between 11°–41° (mIR: 7.5° - 21°) and accordingly in the moderate yellow range. All percentiles of the TS inclination to the right (TSI_r) are between -7° - 15° (mIR: 5° - 10°) and hence in the neutral range. For these lateral movements, the P05 values (-3°–-7°) of most activities account for only half the level as the P95 values (7° - 15°) according to which the TS inclination to the right is higher than to the left.

There is no economic standard available for the LS inclination to the front (LSI_f) and the LS inclination to the right (LSI_r). The percentile values P05 to P95 of the LS inclination to the front are between -20° and 10° (mIR: 5.5° - 9.5°) while the LS inclination to the right is between -10° and - 5° (mIR: 3° - 7.5°). The negative prefix of the LS inclination to the front can be regarded as the tendency of the pelvis to tilt back in a seated position. The higher negative values in PO5 further clarify a higher inclination of the LS to the left.

Most values of the back curvature to the front (BC_f) of the P50, P75 and P95 are in the moderate range (15° - 38°; mIR: 6.5° - 15.5°), while the P05 and P25 values with angles between 8° - 28° are predominantly in the neutral range.

The percentiles P05 and P95 of the back curvature to the right (BC_r) are between -4° - 3°, respectively between 8° - 17° (mIR: 5° - 8°), whereas half of the P95 angles are in the moderate range with 11° - 17°. Exceptions are “palpation” with 8° and “breaks during treatment” with 9°, which are in the neutral range. The comparison of the P05 and P95 values illustrates that the back curvature to the right is a lot stronger at the 95th percentile (positive values) than the back curvature to the left (negative values) of the percentile 05. In addition, the P25-P75 values are almost exclusively in the neutral range and lie between 1° - 14°.

The inclination of the torso to the front (TI_f) shows angle values at P50-95 between -3° and 25° (mIR: 7° - 14.5°), whereby all values (except for P50 and P75 at “breaks during treatments” and P95 at the task “X-ray”) are in the neutral range. For the inclination of the torso to the right (TI_r), all values for P50-P95 lie between 0° - 11° (mIR: 4.5°–9°) and accordingly in the neutral range, except for P95 at the task “X-ray” (moderate range). The observation of the P05 and P95 values reveals a symmetrical angle distribution of these lateral movements.

A similar tendency of the P50-P95 values to be within the neutral range (-6° - 8°; mIR: 5.5° - 10°) can be observed at the back torsion to the right (BT_r). The symmetrical comparison of the angles of P05 and P95 shows a stronger torsion to the right, except for the tasks “impression” (-15°) and “X-ray” (-12°). In these cases, there is a stronger torsion to the left which explains the classification of the values in the moderate range.

Using the OWAS evaluation, the treatment activities show the following distribution of the examined categories of body postures: 50.8 - 92.4 % “sitting”, 6.4 % - 50.9 % “standing” and 0.7 % - 4.2 % “walking”. The back is “straight” from 72.1 % - 85 %, “bowed” from 0.6 % - 1.7 %, 13.8 % - 27.1 % “twisted or inclined to the side” and from 0 % - 0.3 % “bowed and twisted/inclined to the side”.

### Office (II)

The working posture in the category “office” (II) is listed in Additional file 2: Table S2 and comprises all percentiles as well as the modified interquartile range.

In the *head and neck area*, the inclination of the head to the front (HT_f) between P25 and P75 (8° - 24°; mIR: 12° - 15°) is in the neutral range. In P05, the activities are in the neutral range (1° - 2°) (except for “file entries/computer work” -1° in the unfavorable range), whereas the values at P95 between 25° - 31° are almost exclusively in the moderate range. The inclination of the head towards the right (HT_r) has values between -12°–-9° (P5) and 4° - 8° (P95) as well as a mIR of 7°–10°, whereby almost all values of P05-P95 are in a neutral ergonomic range. The symmetrical comparison of the angle values of these two percentiles indicates a more distinctive inclination of the head towards the left due to higher angle values (negative values).

The neck curvature to the front (NC_f) is in the neutral range with -1° - 24° at P50 - P95 (mIR: 13° - 16°). The values of P25 - P95 of the neck curvature to the right (NC_r) are in the neutral range (-8 - 10°; mIR: 7° - 9°). At both movements, nearly all P05 values are in the unfavorable range between -4° and -16° respectively -7–-14° in contrast to the P95 values in the neutral range (14° - 24° respectively 3° - 10°).

It is important to highlight that all tasks of the category office lead to a stronger neck curvature to the left (P05). In the *torso* area, the evaluation parameters TS inclination to the front (TSI_f) at P05-P75 (0° - 19°; mIR: 6° - 12°) is in the neutral range. Almost all values of the percentiles 95 (13° - 29°) are within the moderate range.

With regard to the evaluation parameter TS inclination to the right (TSI_r) (-10° - 8°; mIR: 6° - 7°), back curvature to the right (BC_r) (-2–11°; mIR: 5°–6°), inclination of the torso to the right (TI_r) (-11° - 6°; mIR: 5° - 6°) and back torsion to the right (BT_r) (-10°–6°; mIR: 6° - 7°) almost all percentiles are in the neutral range.

The values P05 and P95 indicate a symmetrical angle distribution at the TS inclination to the side and the inclination of the torso to the side, whereas at the back curvature to the right is asymmetrical with a three-times higher angle value for the curvature to the right compared to the back curvature to the left.

For the back curvature to the front (BC_f), all percentiles of P25-P95 are predominantly in the moderate interval (10° - 41°; mIR: 6° - 12°), except for P05 (neutral range: 7° - 20°). The following tasks need to be classified differently: the activities “model planning” (neutral range: 7° - 18°), P25 “reading patient files (results/tooth model/X-ray)” (neutral range: 20°) as well as P95 “files/computer work” (unfavorable range: 41°). The inclination of the torso to the front (TI_f) shows neutral values in P95 (5° - 15°; mIR: 5° - 9°), whereas the rest of the percentiles of P05 - P75 are predominantly in the unfavorable range. The action “reading patient files (results/tooth model/X-ray)” represents the only exception with values between the 25th and 95th percentile being in the neutral range.

To date, there is no economic standard available for the LS inclination to the front (LSI_f) (-26–2°; mIR: 4° - 9°) and the LS inclination to the right (LSI_r) (-13 - 2°; mIR: 3° - 6°). Due to the high value difference between P05 (-6°–-13°) and P95 (0°–-2°) of the LS inclination to the right, a stronger inclination to the left can be observed.

Based on the OWAS evaluation, the examined office activities show that 46 % - 78.5 % of the activities were performed “sitting”, between 13.1 % - 47.9 % “standing” and 1.5 % - 6 % “walking”. The back is “straight” from 68 % - 83.2 %, “bowed” from 4.4 % - 9.2 %, “twisted or inclined to the side” from 7.2 % - 22.8 % and “bowed and twisted/inclined to the side” from 0.7 % - 5.2 %.

### Other activities (III)

The most important sub-activities of dental treatment belonging to the “other activities category” (Additional file 3: Table S3) are the “conversation” (C), followed by “take/deposit instruments” (In). Therefore, the following description of data focuses on these two activities.

The *head and neck* area with regard to the inclination of the head to the front (HT_f) is in the neutral range at “conversation” between P25-P75 (5° - 16°. mIR: 12°) and “take/deposit instruments” between P05-P25 (6°/17°; mIR: 16°). However, during the task “conversation” P05 (-2°) is in the unfavorable range and P95 (27°) in the moderate range. During the task “take/deposit instruments”, P50 - P95 (27° - 48°) are in the moderate range. With regard to the inclination of the head to the right (HT_r) (C: -12° - 10°, mIR: 9°; In: -16° - 10°, mIR: 10°), almost all P25-P95 values lie in the neutral range, whereas the P05 values are in the unfavorable range. Here, there is a tendency of the head to minimally incline to the left (value of the angles P05 > P95).

The neck curvature to the front (NC_f) is in the optimal range when performing the tasks “conversation” and “take and deposit instruments” at P50 - P95 (C: 0° - 15°, mIR: 12°; In: 2° - 17°, mIR: 13°). P05 and P25 are in the unfavorable range during the tasks “conversation” (-14° respectively -6°) and “take and deposit instruments” (-17°- -6°).

With regard to the evaluation parameter neck curvature to the right (NC_r), the values for “conversation” P25-P95 (-6° - 8°, mIR: 8°) and “take/deposit instruments” P25-P95 (-10° - 8°, mIR: 10°) are in the neutral range. The 5th percentile of both sub-activities is in the unfavorable range (C: -12°; In: -17°). The fact that the angle values of the 5th percentile are higher than those of the 95th percentile indicates a stronger neck curvature to the left.

In the *torso* area, the TS inclination to the front (TSI_f) at P05-P75 for the task “conversation” is in the neutral range (2° - 14°, mIR: 8°) and P95 in the moderate range. During the action “take/deposit instruments”, P05 (7°) and P25 (15°) are in the optimal and P50-P95 (25-47°) in the moderate range with a mIR of 14°.

In both activities, all evaluation parameters of the TS inclination to the right (TSI_r) (C: -6° - 9°, mIR: 6°; In: -7° - 10°, mIR: 7°), back curvature to the right (BC_r) (C: -2° - 10°, mIR: 5°; In: -3° - 11°, mIR: 5°), inclination of the torso to the right (TI_r) (C: -6° - 6°, mIR: 5°; In: -7° - 8°, mIR: 6°) and back torsion to the right (BT_r) (C: -7 - 7°, mIR: 6°; In: -10° - 6°, mIR: 6°) are in the neutral range.

In summarizing assessment of the examined data regarding the preferred direction of movement, it can be said that higher angle values in P95 indicate a TS inclination to the side and back curvature to the right whereas the inclination of the torso and the back torsion to the left and the right are performed to the same extent.

With regard to the back curvature to the front (BC_f), the percentiles P05-P50 (10° - 19°, mIR: 8°) for “conversation” are in the neutral range and the percentiles P75 and P95 in the moderate range. For “take/deposit instruments”, the percentiles P05-P50 (14° - 27°; mIR: 10°) are in the neutral range, whereas P75 has to be assessed as moderate and P95 as awkward.

The inclination of the torso to the front (TI_f) during “conversation” has unfavorable angle values between P05 and P25 (-5° - -2°, mIR: 6°), whereas P50 (1°), P75 (3°) and P95 (10°) can be found in the neutral range. The P05 value of “take/deposit instruments” is in the unfavorable range with -2°. The P25-P75 values (4° - 19°) are in the neutral range. The mIR is 11°. There is no ergonomic standard for the LS inclination to the front (LSI_f) (C: -15° - 0°, mIR: 6°; In: -12° -9°, mIR: 8°) and the LS inclination to the right (LSI_r) (C: -8° - 2°, mIR: 4°; In: -10° 4°, mIR: 6°). With regard to the LS inclination to the side, the comparison between P05 and P95 illustrates that there is a stronger LS inclination to the left during both activities.

The OWAS evaluation for the activity “conversation” based on the *torso* is 5 % “straight”, 3 % “bowed”, 18.6 % “twisted or inclined to the side”, 0.3 % “bowed and twisted/inclined to the side”. Thereby, 48.5 % of the activity was carried out “sitting”, 41 % “standing” and 5 % “walking.” In the activity “take/deposit instruments”, the OWAS evaluation shows 64.7 % a “straight”, 10.5 % a “bowed”, 21.9 % “twisted or inclined to the side” and 2.8 % a “bowed and twisted/inclined to the side” back, 35 % of the activity was completed “standing”, 49.9 % walking and 1 % in a “kneeling” position.

## Discussion

The research objective of this study was to present a comprehensive kinematic analysis of the dentists’ workday in order to analyze whether the high prevalence of musculoskeletal problems is attributable to the body postures during dental tasks per se.

During the dental workdays that have been examined in this study, on an average of 41 % of all tasks can be classified as the treatment of patients. During this treatment of patients, the most common tasks are conducted in seated positions (70 %), whereas in 78 % of all cases a straight back position was held. The fact that the torso is twisted or inclined to the side during 20 % of treatment positions illustrates that there is an asymmetrical body position during treatment.

The average distribution of dental tasks during the examined work routine, which is illustrated in Fig. 2, leads to the verification of hypothesis 1 since the category “treatment” (I) holds the largest share of the dental daily work routine, even though in percentage terms it is only 5 % higher that the category “other activities” (III) which accounts for 36 %. In this context, it is of utmost importance to note that some activities of the category “other activities” (III), for instance “conversations with patients” or “take/deposit instruments” actually belong to category I. Yet, they are not directly related to the treatment but they rather function as accompanying activities for the treatment.

By means of the percentiles, it is possible to make a statement about the joint angles, respectively positions, during the individual segments of the recorded activities in each of the three categories. For the sub-tasks belonging to the category “treatment” (I) between P25-P75 most of the body areas are predominantly in the neutral or moderate range. In particular, extension, flexion and lateral movements of the cervical spine (neck curvature to the front, respectively to the right) during isolated tasks, P05-P25, respectively P75-P95, are in an unfavorable range.

Likewise, unfavorable angle values can be found at the back curvature to the front and the curvature of the torso to the front. The unfavorable angle values are, nonetheless, with up to -7° very small. Despite the ergonomic classification in the unfavorable range, they are rather negligible due to their vicinity to 0° (beginning of neutral classification). In particular, the head tilted to the front and the inclination of the torso to the front are those components of the body posture are a result of the patient treatment activities.. As a basic principle, all joint angles indicate the typical body posture of a dentist during treatment who is either on the right side or behind the dental patient chair [21]: in a seated position without leaning back, the pelvis as well as the entire upper body is anteriorly tilted or inclined to the front, the lumbar spine-thoracic spine area shows a small lateral flexion to the right, whereby the shoulder-neck area is twisted to the left in compensation. The higher angle values of the cervical spine region illustrate the dentist’s inclination to the front while examining the patient’s mouth. In addition, the angle values of P05 and P95 reflect lateral movement to the left (P05) and right (P95). The asymmetrical sitting posture becomes clear due to the different sizes of angle values. The comparison of the angle values of the mIR at treatment (I) 11.5° - 25.5° and those of “office” (II) between 12° - 15°further demonstrates a forced posture with regard to the inclination of the head.

Likewise, the OWAS evaluation confirms that 20 % of the torso posture in the category “treatment” is “twisted or inclined to the side”. This typical treatment position for dentistry to the right of the patient can, in particular, be observed with the right-handed subjects (20 out of 21 participants).

Due to the fact that these movement patterns are adopted several times during the day, the forced postures can occur briefly, dynamically or in a longer static posture. Nonetheless, to what extend the difference between static (longer) and dynamic (brief activity, <4 s.) movements during the individual activities impacts the development of musculoskeletal problems is the subject of a further analysis. Forced postures that are not far from the neutral body and joint angles can possibly lead to problems if they are held statically for longer periods of time [37].

The comparison of the angle values of category II (“office”) with those of category I (“treatment”) shows that values of category II are predominantly in the neutral range. The comparatively high moderate angle values of the back curvature (P25-P95), as well as the unfavorable angles of the inclination of the torso to the front (P05-P75), indicate a hypotonic (kyphotic) sitting posture in which the pelvis is inclined to the back (posterior pelvic tilt) while the thoracic spine area is leaned against the chair (negative angle values). Ellegast et al. [38, 39] have made similar observations regarding the values for the sitting postures at office and monitor-based workplaces. Based on the identical CUELA-System, they register for the 95th percentile values around 25° of the inclination of the head and 10° for the flexion angle of the cervical spine. On average, dentists perform their office tasks at 29°, respectively 17°, in the 95th percentile.

The comparison of these two categories demonstrates the difference between treatment of patients and office work of a dentist. In particular, the differences in the head and cervical spine area illustrate that the body posture during the treatment of patients is worse compared to other activities. This is reflected in higher angles during the dental treatment of patients with an average of 48° (P95 angle of head inclination) or 30° (P 95 angle of cervical spine flexion) compared to 29° (P95 angle of head inclination) and 20 ° (P 95 angle of cervical spine flexion). Hypotheses 2 and 3 can, therefore, be verified as unfavourable posture characteristics for dentists arising especially during treatment in the course of which specific patterns of posture also become apparent. The recording of fine motor movements in the area of the hand and arm was, however, not possible by means of the CUELA-System as the respective sensors are not integrated in the CUELA-System. These activities are nonetheless relevant for the dental profession as precise and delicate work of the practitioner is required to achieve the best possible patient care. The fact that further studies are required to thoroughly analyze these aspects becomes apparent. For example in the surveys of Iranian dental students and Chinese dentists [2, 40], 25 %, respectively 44 % of the respondents suffer from overload and pain in the hands.

Based on previously available published data, it can be assumed that there is a connection between the dental profession and the musculoskeletal problems caused by repetitive movement patterns carried out for several hours daily [3, 10, 17, 41]. Due to the fact that these movement patterns are rather limited because of the specific dental equipment and since a specific body posture is necessary for the optimal patient care, it can be regarded as a forced posture.

Nonetheless, the assessment of the percentile values has to consider the variance of movement as motion patterns are specific to each dentist. This variance of movement (modified interquartile range) illustrates that for many activities the dentist’s postures differ significantly. As a result, the individual-specific postures have to be classified as neutral or even as awkward.

## Conclusion

In summary, as a result of the kinematic analysis several dentist specific postures can be observed. The postures that are adopted show distinct characteristics which are conditioned by the design of the dental work environment. In this context, it can be documented that unfavorable body postures can be predominantly adopted during the treatment rather than to the other examined activities.

In these cases, ergonomically designed dental chairs could significantly improve the body postures. Furthermore, analyses regarding the ergonomic design of dental chairs and dental equipment should be conducted to ensure that dental work can be carried out in neutral body positions. Training in ergonomics should, moreover, be included more intensely in the curriculum of dentistry to prevent musculoskeletal disorders [14, 17, 20]. In German universities, this is often a solitary unit which is integrated into a course. The need of both further analyses and adjustments of the curriculum has been made clear by the present results of the posture analysis, in particular with regard to the head, neck and lumbar spine area when compariing of the activity categories I “treatment” and II “office”.

## Additional files

Additional file 1: Table S1. Treatment: Duration of the respective work stages, percentile values (P05, P25, P50, P75, P95) and values of the modified interquantile range (mIR). (DOCX 40 kb)
Additional file 2: Table S2. Office work and other activities: Duration of the respective work stages, percentile values (P05, P25, P50, P75, P95) and values of the modified interquantile range (mIR). (DOCX 30 kb)
Additional file 3: Table S3. Other activites: Duration of the respective work stages, percentile values (P05, P25, P50, P75, P95) and values of the modified interquantile range (mIR). (DOCX 36 kb)

## Abbreviations

C: Conversation
CUELA-System: Computer-assisted acquisition and long-term analysis of musculoskeletal loads
In: Taking up of instruments / putting down of instruments
mIR: Modified interquantile-range
OWAS: Ovako Working posture Assessment System
P: Percentile
