# Supplementary material for: Kinematic analysis of work-related musculoskeletal loading of trunk among dentists in Germany
Source: BMC Musculoskelet Disord. 2016 Oct 18;17:427. doi: 10.1186/s12891-016-1288-0 (PMC5069924; doi:10.1186/s12891-016-1288-0)
Supplement: Additional file 1: Table S1. — Treatment: Duration of the respective work stages, percentile values (P05, P25, P50, P75, P95) and values of the modified interquantile range (mIR). (DOCX 40 kb) [file 12891_2016_1288_MOESM1_ESM.docx]

**Additional file 1: Table S1.** Treatment: Duration of the respective work stages, percentile values (P05, P25, P50, P75, P95) and values of the modified interquantile range (mIR).

| **Evaluation parameter** | **Activity** | **P05** | **P25** | **P50** | **P75** | **P95** | **mIR** |
| --- | --- | --- | --- | --- | --- | --- | --- |
| **Head tilted to the front (HT_f [°])4** | Impression | 5 | 15 | 25 | 38 | 57 | 26 |
|  | Handicraft activities | 8 | 25 | 37 | 45 | 54 | 23 |
|  | Palpation | 17 | 31 | 35 | 40 | 45 | 14 |
|  | Breaks during treatment | -9 | -2 | 2 | 9 | 23 | 16 |
|  | X-ray | -2 | 10 | 21 | 32 | 47 | 24.5 |
|  | Examination | 9 | 27 | 36 | 43 | 51 | 21 |
|  | Contra-angle/ultrasonic handpiece | 17 | 36 | 45 | 51 | 56 | 19.5 |
| **Head tilted to the right**  **(HT_r [°])** | Impression | -9 | -2 | 3 | 10 | 21 | 15 |
|  | Handicraft activities | -15 | -4 | 4 | 13 | 25 | 20 |
|  | Palpation | -10 | -4 | 3 | 8 | 15 | 12.5 |
|  | Breaks during treatment | -13 | -6 | -1 | 3 | 8 | 10.5 |
|  | X-ray | -17 | -8 | -2 | 6 | 16 | 16.5 |
|  | Examination | -14 | -3 | 8 | 19 | 32 | 23 |
|  | Contra-angle/ultrasonic handpiece | -13 | 0 | 10 | 19 | 29 | 21 |
| **Neck curvature to the front**  **(NC_f [°])** | Impression | 0 | 9 | 16 | 25 | 34 | 17 |
|  | Handicraft activities | -5 | 9 | 17 | 23 | 30 | 17.5 |
|  | Palpation | 9 | 18 | 23 | 26 | 32 | 11.5 |
|  | Breaks during treatment | -19 | -11 | -6 | 1 | 12 | 15.5 |
|  | X-ray | -21 | -6 | 4 | 14 | 22 | 21.5 |
|  | Examination | -3 | 11 | 17 | 23 | 29 | 16 |
|  | Contra-angle/ultrasonic handpiece | 2 | 14 | 20 | 25 | 30 | 14 |
| **Neck curvature to the right (NC_r [°])** | Impression | -10 | -3 | 1 | 6 | 13 | 11.5 |
|  | Handicraft activities | -14 | -5 | 2 | 8 | 17 | 15.5 |
|  | Palpation | -8 | -3 | 2 | 5 | 10 | 9 |
|  | Breaks during treatment | -15 | -8 | -3 | 1 | 5 | 10 |
|  | X-ray | -20 | -11 | -5 | 1 | 11 | 15.5 |
|  | Examination | -15 | -6 | 1 | 9 | 17 | 16 |
|  | Contra-angle/ultrasonic handpiece | -13 | -2 | 5 | 12 | 19 | 16 |
| **TS inclination to the front (TSI_f [°])** | Impression | 1 | 5 | 9 | 15 | 26 | 12.5 |
|  | Handicraft activities | 7 | 14 | 19 | 24 | 30 | 11.5 |
|  | Palpation | 5 | 9 | 11 | 17 | 20 | 7.5 |
|  | Breaks during treatment | 1 | 6 | 9 | 11 | 19 | 9 |
|  | X-ray | -1 | 6 | 17 | 28 | 41 | 21 |
|  | Examination | 7 | 14 | 19 | 22 | 27 | 10 |
|  | Contra-angle/ultrasonic handpiece | 11 | 20 | 25 | 28 | 31 | 10 |
| **TS inclination to the right**  **(TSI_ r [°])** | Impression | -5 | 0 | 2 | 5 | 10 | 7.5 |
|  | Handicraft activities | -5 | 0 | 3 | 6 | 10 | 7.5 |
|  | Palpation | -7 | -1 | 0 | 4 | 8 | 7.5 |
|  | Breaks during treatment | -3 | 1 | 2 | 5 | 7 | 5 |
|  | X-ray | -6 | 0 | 3 | 8 | 14 | 10 |
|  | Examination | -3 | 3 | 7 | 10 | 15 | 9 |
|  | Contra-angle/ultrasonic handpiece | -5 | -2 | 1 | 5 | 9 | 7 |
| **LS inclination to the front (LSI_f [°])** | Impression | -13 | -9 | -7 | -4 | 0 | 6.5 |
|  | Handicraft activities | -14 | -9 | -6 | -3 | 0 | 7 |
|  | Palpation | -20 | -12 | -9 | -6 | -4 | 8 |
|  | Breaks during treatment | -17 | -15 | -14 | -11 | -3 | 7 |
|  | X-ray | -9 | -4 | 0 | 4 | 10 | 9.5 |
|  | Examination | -13 | -9 | -7 | -5 | -2 | 5.5 |
|  | Contra-angle/ultrasonic handpiece | -14 | -10 | -7 | -5 | -3 | 5.5 |
| **LS inclination to the right (LSI_r [°])** | Impression | -8 | -5 | -3 | -2 | 1 | 4.5 |
|  | Handicraft activities | -7 | -5 | -3 | -1 | 2 | 4.5 |
|  | Palpation | -5 | -3 | -2 | 0 | 1 | 3 |
|  | Breaks during treatment | -6 | -3 | -1 | 0 | 3 | 4.5 |
|  | X-ray | -10 | -6 | -3 | 0 | 5 | 7.5 |
|  | Examination | -8 | -6 | -4 | -2 | 1 | 4.5 |
|  | Contra-angle/ultrasonic handpiece | -7 | -4 | -3 | -2 | 1 | 4 |
| **Back curvature to the front (BC_f [°])** | Impression | 8 | 12 | 15 | 21 | 30 | 11 |
|  | Handicraft activities | 15 | 21 | 25 | 30 | 35 | 10 |
|  | Palpation | 16 | 19 | 21 | 25 | 29 | 6.5 |
|  | Breaks during treatment | 11 | 18 | 22 | 25 | 28 | 8.5 |
|  | X-ray | 3 | 10 | 18 | 26 | 34 | 15.5 |
|  | Examination | 16 | 22 | 25 | 28 | 33 | 8.5 |
|  | Contra-angle/ultrasonic handpiece | 22 | 28 | 32 | 35 | 38 | 8 |
| **Back curvature to the right (BC_r [°])** | Impression | -1 | 3 | 6 | 8 | 11 | 6 |
|  | Handicraft activities | -1 | 3 | 6 | 8 | 11 | 6 |
|  | Palpation | -4 | 1 | 3 | 5 | 8 | 6 |
|  | Breaks during treatment | -1 | 2 | 3 | 6 | 9 | 5 |
|  | X-ray | -1 | 3 | 6 | 10 | 15 | 8 |
|  | Examination | 3 | 7 | 11 | 14 | 17 | 7 |
|  | Contra-angle/ultrasonic handpiece | 2 | 5 | 7 | 9 | 12 | 5 |
| **Inclination of the torso to the front (TI_f [°])** | Impression | -5 | -2 | 1 | 6 | 12 | 8.5 |
|  | Handicraft activities | -2 | 3 | 7 | 10 | 14 | 8 |
|  | Palpation | -6 | -1 | 1 | 5 | 8 | 7 |
|  | Breaks during treatment | -7 | -4 | -3 | -1 | 7 | 7 |
|  | X-ray | -4 | 2 | 8 | 16 | 25 | 14.5 |
|  | Examination | -3 | 3 | 6 | 9 | 12 | 7.5 |
|  | Contra-angle/ultrasonic handpiece | -1 | 6 | 9 | 11 | 13 | 7 |
| **Inclination of the torso to the right (TI_r [°])** | Impression | -6 | -2 | 0 | 3 | 7 | 6.5 |
|  | Handicraft activities | -5 | -1 | 1 | 3 | 7 | 6 |
|  | Palpation | -6 | -2 | 0 | 3 | 6 | 6 |
|  | Breaks during treatment | -3 | 0 | 1 | 3 | 6 | 4.5 |
|  | X-ray | -7 | -1 | 2 | 6 | 11 | 9 |
|  | Examination | -4 | 0 | 4 | 6 | 10 | 7 |
|  | Contra-angle/ultrasonic handpiece | -4 | 0 | 2 | 4 | 7 | 5.5 |
| **Back torsion to the right (BT_r [°])** | Impression | -15 | -9 | -6 | -2 | 3 | 9 |
|  | Handicraft activities | -9 | -5 | -3 | 0 | 7 | 8 |
|  | Palpation | -8 | -5 | -2 | 1 | 3 | 5.5 |
|  | Breaks during treatment | -4 | 0 | 2 | 4 | 7 | 5.5 |
|  | X-ray | -12 | -5 | -1 | 3 | 8 | 10 |
|  | Examination | -7 | -4 | -1 | 1 | 7 | 7 |
|  | Contra-angle/ultrasonic handpiece | -6 | -4 | -3 | -1 | 5 | 5.5 |
