# Supplementary material for: Kinematic analysis of work-related musculoskeletal loading of trunk among dentists in Germany
Source: BMC Musculoskelet Disord. 2016 Oct 18;17:427. doi: 10.1186/s12891-016-1288-0 (PMC5069924; doi:10.1186/s12891-016-1288-0)
Supplement: Additional file 2: Table S2. — Office work and other activities: Duration of the respective work stages, percentile values (P05, P25, P50, P75, P95) and values of the modified interquantile range (mIR). (DOCX 30 kb) [file 12891_2016_1288_MOESM2_ESM.docx]

**Additional file 2: Table S2.** Office work and other activities: Duration of the respective work stages, percentile values (P05, P25, P50, P75, P95) and values of the modified interquantile range (mIR).

| **Evaluation parameter** | **Activity** | **P05** | **P25** | **P50** | **P75** | **P95** | **mIR** |
| --- | --- | --- | --- | --- | --- | --- | --- |
| **Office Work (II)** | | | | | | | |
| **Head tilted to the front (HT_f) [°]** | Modell planning | 1 | 9 | 17 | 24 | 31 | 15 |
|  | Phone calls | 1 | 8 | 13 | 18 | 25 | 12 |
|  | Files/computer work | -1 | 8 | 14 | 20 | 29 | 15 |
|  | Reading patient files (results/tooth model/X-ray) | 2 | 11 | 17 | 23 | 31 | 14 |
| **Head tilted to the right (HT_r) [°]** | Modell planning | -9 | -5 | -2 | 1 | 5 | 7 |
|  | Phone calls | -10 | -5 | -2 | 0 | 4 | 7 |
|  | Files/computer work | -12 | -6 | -2 | 1 | 8 | 10 |
|  | Reading patient files (results/tooth model/X-ray) | -12 | -6 | -2 | 1 | 7 | 9 |
| **Neck curvature to the front (NC_f [°])** | Modell planning | -4 | 4 | 11 | 17 | 24 | 14 |
|  | Phone calls | -10 | -2 | 2 | 8 | 16 | 13 |
|  | Files/computer work | -16 | -7 | 0 | 7 | 15 | 16 |
|  | Reading patient files (results/tooth model/X-ray) | -16 | -7 | -1 | 5 | 14 | 15 |
| **Neck curvature to the right (NC_r [°])** | Modell planning | -7 | -3 | 0 | 4 | 10 | 9 |
|  | Phone calls | -11 | -7 | -4 | -1 | 3 | 7 |
|  | Files/computer work | -12 | -7 | -3 | 1 | 6 | 9 |
|  | Reading patient files (results/tooth model/X-ray) | -14 | -8 | -4 | -1 | 5 | 9 |
| **TS inclination to the front (TSI_f [°])** | Modell planning | 0 | 3 | 5 | 9 | 13 | 6 |
|  | Phone calls | 0 | 5 | 10 | 15 | 22 | 11 |
|  | Files/computer work | 3 | 10 | 14 | 18 | 24 | 10 |
|  | Reading patient files (results/tooth model/X-ray) | 5 | 14 | 19 | 23 | 29 | 12 |
| **TS inclination to the right (TSI_r [°])** | Modell planning | -10 | -5 | -2 | 0 | 3 | 7 |
|  | Phone calls | -5 | -1 | 2 | 4 | 7 | 6 |
|  | Files/computer work | -5 | -1 | 1 | 4 | 7 | 6 |
|  | Reading patient files (results/tooth model/X-ray) | -4 | 0 | 2 | 4 | 8 | 6 |
| **LS inclination to the front (LSI_f [°])** | Modell planning | -10 | -9 | -7 | -5 | -2 | 4 |
|  | Phone calls | -26 | -23 | -19 | -16 | -7 | 9 |
|  | Files/computer work | -25 | -22 | -18 | -14 | -9 | 8 |
|  | Reading patient files (results/tooth model/X-ray) | -13 | -8 | -5 | -2 | 2 | 7 |
| **LS inclination to the right (LSI_r [°])** | Modell planning | -13 | -6 | -4 | -2 | 0 | 6 |
|  | Phone calls | -6 | -3 | -2 | 0 | 2 | 4 |
|  | Files/computer work | -7 | -5 | -3 | -2 | 0 | 3 |
|  | Reading patient files (results/tooth model/X-ray) | -8 | -5 | -3 | -1 | 2 | 5 |
| **Back curvature to the front (BC_f [°])** | Modell planning | 7 | 10 | 13 | 15 | 18 | 6 |
|  | Phone calls | 15 | 23 | 30 | 35 | 39 | 12 |
|  | Files/computer work | 20 | 27 | 33 | 37 | 41 | 10 |
|  | Reading patient files (results/tooth model/X-ray) | 13 | 20 | 24 | 27 | 32 | 9 |
| **Back curvature to the right (BC_r [°])** | Modell planning | -2 | 0 | 1 | 4 | 9 | 6 |
|  | Phone calls | -2 | 1 | 3 | 5 | 8 | 5 |
|  | Files/computer work | 0 | 3 | 5 | 7 | 10 | 5 |
|  | Reading patient files (results/tooth model/X-ray) | -1 | 3 | 5 | 7 | 11 | 6 |
| **Inclination of the torso to the front (TI_f [°])** | Modell planning | -4 | -2 | -1 | 2 | 5 | 5 |
|  | Phone calls | -11 | -8 | -5 | -1 | 5 | 8 |
|  | Files/computer work | -10 | -5 | -2 | 1 | 6 | 8 |
|  | Reading patient files (results/tooth model/X-ray) | -3 | 3 | 7 | 10 | 15 | 9 |
| **Inclination of the torso to the right (TI_r [°])** | Modell planning | -11 | -5 | -3 | -1 | 2 | 6 |
|  | Phone calls | -5 | -1 | 1 | 2 | 5 | 5 |
|  | Files/computer work | -5 | -2 | 0 | 2 | 5 | 5 |
|  | Reading patient files (results/tooth model/X-ray) | -4 | -1 | 1 | 3 | 6 | 5 |
| **Back torsion to the right (BT_r [°])** | Modell planning | -10 | -4 | -3 | -1 | 3 | 6 |
|  | Phone calls | -6 | -3 | -1 | 2 | 5 | 6 |
|  | Files/computer work | -7 | -3 | 0 | 3 | 6 | 7 |
|  | Reading patient files (results/tooth model/X-ray) | -7 | -4 | -1 | 1 | 6 | 6 |
