# Supplementary material for: Kinematic analysis of work-related musculoskeletal loading of trunk among dentists in Germany
Source: BMC Musculoskelet Disord. 2016 Oct 18;17:427. doi: 10.1186/s12891-016-1288-0 (PMC5069924; doi:10.1186/s12891-016-1288-0)
Supplement: Additional file 3: Table S3. — Other activites: Duration of the respective work stages, percentile values (P05, P25, P50, P75, P95) and values of the modified interquantile range (mIR). (DOCX 36 kb) [file 12891_2016_1288_MOESM3_ESM.docx]

**Additional file 3: Table S3.** Other activites: Duration of the respective work stages, percentile values (P05, P25, P50, P75, P95) and values of the modified interquantile range (mIR).

| **Other Activites (III)** | | | | | | | |
| --- | --- | --- | --- | --- | --- | --- | --- |
| **Head tilted to the front**  **(HT_f) [°]** | Meeting | -1 | 6 | 10 | 17 | 27 | 11 |
|  | Conversation | -2 | 5 | 10 | 16 | 27 | 12 |
|  | Hygiene | 0 | 9 | 16 | 23 | 32 | 12 |
|  | Take/deposit instrument | 6 | 17 | 27 | 38 | 48 | 16 |
|  | Laboratory | 3 | 17 | 25 | 32 | 40 | 15 |
|  | Walk | -2 | 5 | 11 | 18 | 30 | 12 |
| **Head tilted to the right (HT_r) [°]** | Meeting | -13 | -6 | -3 | 1 | 8 | 9 |
|  | Conversation | -12 | -5 | -1 | 3 | 10 | 9 |
|  | Hygiene | -12 | -6 | -2 | 3 | 10 | 9 |
|  | Take/deposit instrument | -16 | -9 | -4 | 2 | 10 | 10 |
|  | Laboratory | -17 | -11 | -6 | -2 | 6 | 9 |
|  | Walk | -13 | -6 | -2 | 3 | 10 | 9 |
| **Neck curvature to the front (NC_f [°])** | Meeting | -14 | -7 | -1 | 4 | 12 | 10 |
|  | Conversation | -14 | -6 | 0 | 6 | 15 | 12 |
|  | Hygiene | -11 | -2 | 4 | 10 | 17 | 11 |
|  | Take/deposit instrument | -17 | -6 | 2 | 10 | 17 | 13 |
|  | Laboratory | -12 | 0 | 7 | 13 | 22 | 13 |
|  | Walk | -11 | -4 | 2 | 9 | 17 | 11 |
| **Neck curvature to the right (NC_r [°])** | Meeting | -7 | 1 | 6 | 9 | 13 | 8 |
|  | Conversation | -12 | -6 | -2 | 2 | 8 | 8 |
|  | Hygiene | -12 | -7 | -3 | 1 | 8 | 8 |
|  | Take/deposit instrument | -17 | -10 | -4 | 1 | 8 | 10 |
|  | Laboratory | -17 | -11 | -6 | -2 | 5 | 9 |
|  | Walk | -14 | -7 | -3 | 1 | 8 | 9 |
| **TS inclination to the front (TSI_f [°])** | Meeting | 4 | 8 | 14 | 16 | 21 | 7 |
|  | Conversation | 2 | 7 | 10 | 14 | 22 | 8 |
|  | Hygiene | 2 | 7 | 11 | 16 | 25 | 9 |
|  | Take/deposit instrument | 7 | 15 | 25 | 36 | 47 | 14 |
|  | Laboratory | 6 | 13 | 17 | 22 | 30 | 10 |
|  | Walk | 1 | 4 | 8 | 13 | 24 | 9 |
| **TS inclination to the right (TSI_r [°])** | Meeting | -14 | -11 | -10 | -6 | 6 | 8 |
|  | Conversation | -6 | -1 | 1 | 4 | 9 | 6 |
|  | Hygiene | -7 | -1 | 2 | 4 | 9 | 7 |
|  | Take/deposit instrument | -7 | -2 | 1 | 4 | 10 | 7 |
|  | Laboratory | -6 | -3 | -1 | 2 | 7 | 5 |
|  | Walk | -8 | -2 | 2 | 6 | 12 | 8 |
| **LS inclination to the front (LSI_f [°])** | Meeting | -44 | -42 | -31 | -29 | -19 | 11 |
|  | Conversation | -15 | -11 | -9 | -6 | 0 | 6 |
|  | Hygiene | -13 | -9 | -6 | -3 | 3 | 6 |
|  | Take/deposit instrument | -12 | -7 | -3 | 2 | 9 | 8 |
|  | Laboratory | -12 | -9 | -6 | -3 | 4 | 6 |
|  | Walk | -8 | -4 | -1 | 2 | 8 | 6 |
| **LS inclination to the right (LSI_r [°])** | Meeting | -7 | -5 | -4 | -3 | 1 | 3 |
|  | Conversation | -8 | -5 | -3 | -1 | 2 | 4 |
|  | Hygiene | -8 | -5 | -3 | -1 | 3 | 5 |
|  | Take/deposit instrument | -10 | -6 | -3 | 0 | 4 | 6 |
|  | Laboratory | -8 | -6 | -4 | -2 | 2 | 4 |
|  | Walk | -10 | -6 | -3 | 1 | 5 | 6 |
| **Back curvature to the front (BC_f [°])** | Meeting | 33 | 43 | 45 | 52 | 56 | 8 |
|  | Conversation | 10 | 15 | 19 | 23 | 29 | 8 |
|  | Hygiene | 10 | 14 | 17 | 21 | 27 | 7 |
|  | Take/deposit instrument | 14 | 21 | 27 | 35 | 42 | 10 |
|  | Laboratory | 11 | 18 | 24 | 29 | 34 | 9 |
|  | Walk | 3 | 6 | 9 | 13 | 20 | 7 |
| **Back curvature to the right (BC_r [°])** | Meeting | -11 | -8 | -5 | -1 | 7 | 7 |
|  | Conversation | -2 | 2 | 4 | 7 | 10 | 5 |
|  | Hygiene | -2 | 2 | 4 | 7 | 10 | 5 |
|  | Take/deposit instrument | -3 | 1 | 4 | 7 | 11 | 5 |
|  | Laboratory | -1 | 1 | 3 | 6 | 10 | 4 |
|  | Walk | -3 | 1 | 4 | 7 | 12 | 6 |
| **Inclination of the torso to the front (TI_f [°])** | Meeting | -19 | -16 | -9 | -7 | 0 | 8 |
|  | Conversation | -5 | -2 | 1 | 3 | 10 | 6 |
|  | Hygiene | -4 | -1 | 2 | 6 | 13 | 7 |
|  | Take/deposit instrument | -2 | 4 | 11 | 19 | 27 | 11 |
|  | Laboratory | -1 | 3 | 6 | 8 | 14 | 6 |
|  | Walk | -3 | 1 | 3 | 7 | 15 | 7 |
| **Inclination of the torso to the right (TI_r [°])** | Meeting | -10 | -8 | -7 | -5 | 3 | 6 |
|  | Conversation | -6 | -2 | 0 | 2 | 6 | 5 |
|  | Hygiene | -7 | -2 | 0 | 3 | 7 | 6 |
|  | Take/deposit instrument | -7 | -3 | 0 | 3 | 8 | 6 |
|  | Laboratory | -6 | -3 | -1 | 1 | 5 | 4 |
|  | Walk | -8 | -3 | 1 | 4 | 10 | 7 |
| **Back torsion to the right (BT_r [°])** | Meeting | -5 | 3 | 6 | 10 | 12 | 7 |
|  | Conversation | -7 | -3 | 0 | 3 | 7 | 6 |
|  | Hygiene | -6 | -2 | 1 | 4 | 8 | 6 |
|  | Take/deposit instrument | -10 | -6 | -3 | 1 | 6 | 6 |
|  | Laboratory | -6 | -2 | 0 | 2 | 5 | 5 |
|  | Walk | -10 | -4 | 0 | 3 | 9 | 7 |
